# Peripheral polyneuropathy from electrodiagnostic tests: a 10-year etiology and neurophysiology overview

**DOI:** 10.1590/0004-282X-ANP-2020-0561

**Published:** 2021-11-30

**Authors:** Renata Dal-Prá Ducci, Camila Lorenzini Tessaro, Cláudia Suemi Kamoi Kay, Otto Jesus Hernandez Fustes, Lineu Cesar Werneck, Paulo José Lorenzoni, Rosana Herminia Scola

**Affiliations:** 1 Universidade Federal do Paraná, Hospital de Clínicas, Departamento de Medicina Interna, Divisão de Neurologia, Serviço de Doenças Neuromusculares, Curitiba PR, Brazil. Universidade Federal do Paraná Hospital de Clínicas Departamento de Medicina Interna Curitiba PR Brazil; 2 Universidade Federal do Paraná, Curitiba PR, Brazil. Universidade Federal do Paraná Curitiba PR Brazil

**Keywords:** Polyneuropathies, Epidemiology, Diagnosis, Electrodiagnosis, Polineuropatias, Epidemiologia, Diagnóstico, Eletrodiagnóstico

## Abstract

**Background:**

Polyneuropathies are characterized by a symmetrical impairment of the peripheral nervous system, resulting in sensory, motor and/or autonomic deficits. Due to the heterogeneity of causes, an etiological diagnosis for polyneuropathy is challenging.

**Objective:**

The aim of this study was to determine the main causes of polyneuropathy confirmed by electrodiagnostic (EDX) tests in a tertiary service and its neurophysiological aspects.

**Methods:**

This observational cross-sectional study from a neuromuscular disorders center included individuals whose electrodiagnostic tests performed between 2008 and 2017 confirmed a diagnosis of polyneuropathy. Through analysis of medical records, polyneuropathies were classified according to etiology and neurophysiological aspect.

**Results:**

Of the 380 included patients, 59.5% were male, with a median age of 43 years. The main etiologies were: inflammatory (23.7%), hereditary (18.9%), idiopathic (13.7%), multifactorial (11.1%), and diabetes (10.8%). The main electrophysiological patterns were axonal sensorimotor polyneuropathy (36.1%) and “demyelinating and axonal” sensorimotor polyneuropathy (27.9%). Axonal patterns showed greater etiological heterogeneity, with a predominance of idiopathic and multifactorial polyneuropathy, while demyelinating and “demyelinating and axonal” polyneuropathies had a significantly fewer etiologies, with a predominance of hereditary and inflammatory polyneuropathies.

**Conclusion:**

The main causes of polyneuropathy confirmed by EDX test in this study were those that presented a severe, atypical and/or rapidly progressing pattern. Other causes were hereditary and those that defy clinical reasoning, such as multiple risk factors; some polyneuropathies did not have a specific etiology. EDX tests are useful for etiological diagnosis of rare polyneuropathies, because neurophysiological patterns are correlated with specific etiologies.

## RESUMO

**Antecedentes:**

As polineuropatias caracterizam-se por um acometimento simétrico do sistema nervoso periférico, resultando em alterações sensitivas, motoras e/ou autonômicas. Devido à heterogeneidade de causas, estabelecer um diagnóstico etiológico para polineuropatia é desafiador.

**Objetivo:**

O objetivo desse estudo foi determinar as principais causas de polineuropatia confirmada pela eletroneuromiografia em um serviço terciário e seus aspectos neurofisiológicos.

**Métodos:**

Esse estudo transversal observacional de um centro de doenças neuromusculares incluiu indivíduos cuja eletroneuromiografia, realizada entre 2008 e 2017, estabeleceu o diagnóstico de polineuropatia. Através da análise dos prontuários, as polineuropatias foram classificadas de acordo com a etiologia e aspecto neurofisiológico.

**Resultados:**

Dos 380 pacientes incluídos, 59,5% eram homens, com uma mediana de idade de 43 anos. As principais etiologias foram: imunomediada (23,7%), hereditária (18,9%), idiopática (13,7%), multifatorial (11,1%) e diabetes (10,8%). Os principais padrões neurofisiológicos foram polineuropatia axonal sensitivo-motora (36,1%) e polineuropatia mista sensitivo-motora (27,9%). Os padrões axonais demonstraram maior heterogeneidade etiológica, com um predomínio das polineuropatias idiopática e multifatorial, enquanto os padrões desmielinizantes e mistos apresentaram uma significativa restrição de etiologias, com predomínio das polineuropatias hereditária e imunomediada.

**Conclusão:**

As principais causas de polineuropatia confirmadas pela eletroneuromiografia nesse estudo foram aquelas que apresentaram um padrão de acometimento grave, atípico e/ou rapidamente progressivo, além das polineuropatias hereditárias e daquelas que desafiam o raciocínio clínico, caracterizadas pela presença múltiplos fatores de risco ou pela ausência de uma etiologia. A eletroneuromiografia é útil para o diagnóstico etiológico de polineuropatias raras, já que os padrões neurofisiológicos se correlacionam com etiologias específicas.

## INTRODUCTION

Polyneuropathies are characterized by a symmetrical and diffuse impairment of the peripheral nervous system, which may affect motor, sensitive or autonomic nerve fibers. Clinical features vary widely and include muscle weakness and atrophy, paresthesia, pain, hypoesthesia and autonomic symptoms^1^^,^^2^. Polyneuropathies have a great heterogeneity of causes. Diabetes mellitus, alcohol abuse, genetic conditions, nutritional deficiency, drug toxicity, autoimmunity, infection, and malignancy are some examples^2^^,^^3^. Worldwide, especially in developed countries, the main etiology of peripheral polyneuropathy is diabetes mellitus, with a prevalence of 30 to 66%^4^^,^^5^. Therefore, an etiological diagnosis of polyneuropathy is a challenge that demands time and financial resources^3^. Even with appropriate evaluation, between 20 and 30% of polyneuropathies remain without a definite cause^1^^,^^2^. 

Electrodiagnostic (EDX) testing, which include nerve conduction studies and needle electromyography, is a method to measure the electrical activity of the peripheral nervous system that is considered an extension of the neurological exam in the evaluation of polyneuropathies^3^^,^^4^. Several publications have discussed the indications of this exam in the diagnosis of neuropathies^6^^-^^8^. Unfortunately, EDX testing is not used by all physicians who treat patients with polyneuropathy. The American Association of Neuromuscular and Electrodiagnostic Medicine (AANEM) states in its official position in 2017 that EDX testing should be considered in the following cases: when no cause has been identified; in severe cases; in atypical presentations; in the presence of signs or symptoms suggestive of another neuromuscular disease; when there is a positive family history of hereditary neuropathy; or when there is a history of exposure to toxic substances known to cause polyneuropathy^9^.

 Due to discussions regarding the indication of electrodiagnostic tests and due to the lack of information related to the epidemiology of polyneuropathies^3^^,^^10^, the aim of this study was to determine the main etiologies of polyneuropathy confirmed by electrodiagnostic tests within a single specialized tertiary center of Southern Brazil. The main clinical manifestations, risk factors, and associated electrophysiological findings are reported.

## METHODS

**Figure 1.**
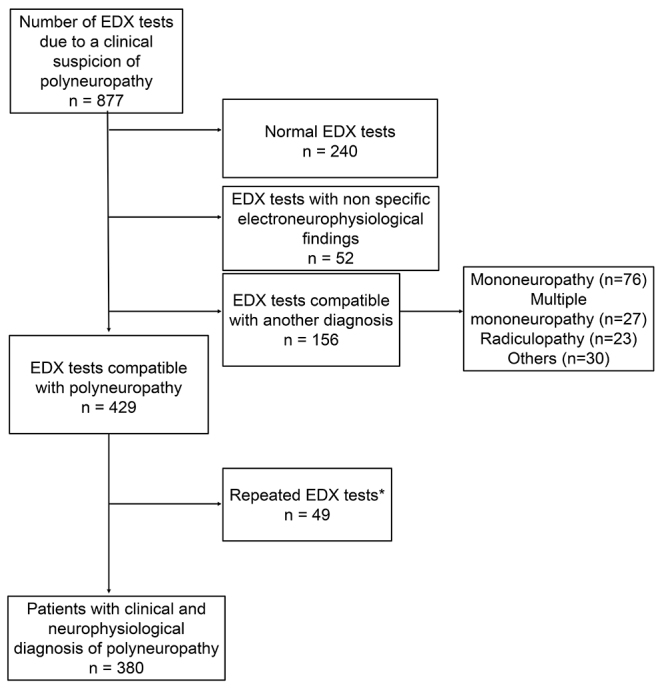
Flow chart of the study methodology.

We selected all patients with a clinical suspicion of polyneuropathy (symptoms and signs) who attended the neuromuscular disorder center at Hospital de Clínicas da Universidade Federal do Paraná (Curitiba, Brazil) between 2008 and 2017 and who underwent EDX testing. The diagnosis of polyneuropathy is usually done by the combination of clinical and electroneurophysiological features^11^. In this study, we included patients whose EDX test confirmed polyneuropathy and excluded patients whose EDX test was normal, with nonspecific electroneurophysiological findings, or showed another diagnosis, such as radiculopathy, mononeuropathy and multiple mononeuropathy. Figure 1 shows the criteria used for selecting the patients. The study was approved by the institution's Ethics Committee (Comitê de Ética em Pesquisa em Seres Humanos).

*EDX tests performed in the investigation of the same patient. EDX: Electrodiagnostic test.

In our hospital, patients with suspected polyneuropathy are determined by the examination of the neurologist. Nerve conduction studies and needle electromyography (EMG) are performed in all patients according to standard protocols^11^^,^^12^. Reference values of the nerve conduction studies are based on previously published standard protocols^12^.

The electrophysiological criterion used for definition of polyneuropathy was an abnormality in at least one parameter in two or more peripheral nerves of two or more extremities^11^^,^^13^. All EDX tests were retrospectively reviewed by the authors to verify the fulfillment of this criterion. 

Electrophysiological criteria for axonal polyneuropathies were: reduced amplitude of sensory nerve action potential (SNAP) and/or compound muscle action potential (CMAP), with normal or only slightly slowed nerve conduction velocity (NCV), distal motor latency (DML). and late responses. These findings may be associated with denervation and/or reinervation signs at EMG. Isolated signs of denervation were also used as criteria for axonal polyneuropathy^12^^,^^14^. Criteria for demyelinating polyneuropathies were: prolonged DML (> 130% of the upper limit of normal), slowed NCV (< 70% of the lower limit of normal), and/or prolonged or absent late responses (> 130% of the upper limit of normal), with a normal amplitude of SNAP and CMAP and normal results of EMG^12^^,^^14^. Polyneuropathies were considered “demyelinating and axonal” when there were demyelinating findings, but also reduced amplitudes and evidence of denervation, or when there were axonal criteria but with conduction velocity below 70% of the lower limit of normality^13^. 

Polyneuropathies were classified as motor or sensory if disturbances were present only in motor or sensitive nerve fibers, respectively. Sensorimotor polyneuropathy was defined by abnormalities involving motor and sensitive nerve fibers^12^.

Considering that only patients with electrodiagnostic test compatible with polyneuropathy were included, this study did not evaluate small fiber polyneuropathies, which have as a diagnostic criterion a normal EDX test^15^^,^^16^.

A retrospective analysis of medical records was performed, collecting data on symptoms and signs, risk factors for neuropathy, routine and additional laboratory tests, and complementary tests. Routine tests included complete blood count, fasting blood glucose, glycated hemoglobin (Hb1Ac), thyroid stimulating hormone (TSH), vitamin B12, folic acid, creatinine, urea, alanine aminotransferase (ALT), aspartate aminotransferase (AST), direct and indirect total bilirubin, albumin, alkaline phosphatase, gamma-glutamyl transferase, erythrocyte sedimentation rate (ESR), C-reactive protein (CRP), serology for human immunodeficiency virus (HIV), hepatitis B and C, syphilis, and lymph node acid-fast bacilli (AFB). Research on antinuclear antibodies (ANA), rheumatoid factor (RF), anti-Ro and anti-La antibodies, cerebrospinal fluid (CSF) analysis, investigation of malignancy, and genetic tests were considered additional exams^3^^,^^10^. Although this was a retrospective study, in which the difficulty of getting information is to be expected, no patient was excluded because of insufficient data.

The patients were grouped according to the number of polyneuropathy etiologies as monocausal, multifactorial, or idiopathic. After evaluation of collected variables, the etiology of the polyneuropathies was determined retrospectively according to the following diagnostic criteria:

- Hereditary: considered in the presence of a confirmatory genetic test or in the presence of a positive family history and/or highly suggestive clinical condition, when other causes were excluded^17^.
- Diabetes: considered in individuals with type 1 or type 2 diabetes mellitus using hypoglycemic agents, and/or in those with fasting glucose greater than or equal to 126 mg/dL or Hb1Ac above or equal to 6.5%, confirmed at two strengths^18^.
- Guillain-Barré syndrome (GBS): diagnosed according to the diagnostic criteria of Asbury and Cornblath, which include progressive upper and lower limb weakness, global areflexia, a progressive phase lasting a maximum of 4 weeks, albumin-cytological dissociation in CSF and nerve conduction changes in EDX testing, whether demyelinating (acute inflammatory demyelinating polyneuropathy [AIDP]) or axonal (acute motor axonal neuropathy [AMAN] or acute sensory motor axonal neuropathy [AMSAN]). The GBS variant, Miller-Fischer syndrome, was diagnosed in the presence of ophthalmoplegia, ataxia, and global areflexia^19^.
- Chronic inflammatory demyelinating polyneuropathy (CIDP): symmetrical progressive distal and proximal weakness associated with sensory changes in extremities, with a progressive phase lasting at least 8 weeks, global areflexia or global hyporeflexia, neurophysiological findings compatible with demyelination, and albumin-cytological dissociation in CSF. The most common atypical form of CIDP, Lewis-Sumner syndrome, was considered in the presence of an asymmetrical clinical picture, with predominance in upper limbs^20^.
- Alcohol: in the presence of reported alcohol abuse, based on the amount and time of consumption and the absence of another cause that better explained symptoms^21^.
- Vitamin B12 deficiency: serum vitamin B12 level below 350 pg/mL^22^ was indispensable.
- Toxicity (by medication or other substances): considered in the presence of current or past use of a neurotoxic drug or a history of prolonged contact with a toxic substance, in addition to finding a temporal relationship between the onset of use/contact and the onset of symptoms and/or improvement or stabilization of symptoms after withdrawal of the offending agent^23^.
- Infectious: in the presence of a previous diagnosis of any infectious disease known to cause polyneuropathy (e.g. leprosy, HIV, hepatitis B and C), current or previous treatment for any of these infections, and/or laboratory or skin biopsy changes suggestive of an infectious disease associated with polyneuropathy^24^.
- Vasculitis: presence of a systemic disease known to be associated with vasculitis and/or in the presence of vascular changes compatible with vasculitis on nerve biopsy^25^.
- Paraneoplastic: considered when primary neoplasia was diagnosed during the investigation, even without onconeural antibody tests, or in those patients at high risk of having an associated neoplasia, always in the absence of other causes or risk factors^26^.

Finally, individuals with no risk factors for polyneuropathy or altered laboratory tests made up the group of idiopathic polyneuropathies. Those who underwent all routine laboratory tests were classified as “idiopathic polyneuropathy with complete basic investigation” ^5^. On the other hand, if routine examinations were not performed or were only partially performed and in the absence of any risk factors, polyneuropathy was considered “idiopathic with incomplete basic investigation”. Individuals with various risk factors for polyneuropathy, with no clear predominance of either factor, were classified as “multifactorial polyneuropathy”^27^. It is important to highlight that we could not define how much each etiologic factor contributed to the pathophysiology of polyneuropathy. Thus, patients who met this criterion had a probable multifactorial etiology rather than a definite one.

The above criteria were used retrospectively. That is, the etiological diagnosis previously defined in the medical record was reviewed, applying the study criteria. However, the etiology of polyneuropathy was not changed.

The electrophysiological pattern of the EDX test was also used to classify the patients. Demographic characteristics and main etiologies of polyneuropathy for each electrophysiological pattern of the selected patients were determined for each group.

From collected data, a descriptive analysis of all variables was performed. Thus, it was possible to evaluate the frequency of qualitative variables and the symmetry of the distributions of quantitative variables (normal distribution assessed by the Shapiro-Wilk test). Quantitative variables were described by median (minimum -maximum range) because the data did not always have a normal distribution, while qualitative variables were described by frequency and percentage.

## RESULTS

Of 877 EDX tests performed on a clinical suspicion of polyneuropathy, 429 (48.9%) confirmed this diagnosis. Because some individuals underwent EDX test more than once, we identified 380 patients whose EDX test was compatible with polyneuropathy. The sample population was predominantly male (59.5%), with a male-to-female ratio of 3:2. The median age was 43 years (range 0.3-85). The median time to onset of symptoms before the first appointment was 2 years (range 0-58).

The risk factors, symptoms, and signs of investigated individuals are shown in Table 1. The main risk factors were diabetes mellitus (23.2%) and alcoholism (14.2%). The symptoms reported more often were distal weakness in the lower limbs (65.8%) and paresthesia (49.2%). The main neurological signs detected were sensory disturbance (72.9%), muscle weakness (72.9%), and areflexia (61.6%). 

**Table 1.** Distribution of risk factors and neurological symptoms and signs in 380 patients whose EDX test indicated polyneuropathy.

Variable		n (%)
Risk factor	Diabetes	88 (23.2)
	Alcohol abuse	54 (14.2)
	Family history	46 (11.8)
	Toxic medication^a^	43 (11.3)
	Hypothyroidism	30 (7.9)
	Previous history of malignancy	27 (7.1)
	Exposure to toxic substances^b^	20 (5.3)
	HIV	10 (2.6)
	Hepatitis^c^	10 (2.6)
Neurological symptoms	Distal weakness in the lower limbs	250 (65.8)
	Paresthesia	187 (49.2)
	Distal weakness in the upper limbs	167 (43.9)
	Neuropathic pain	132 (34.7)
	Hypoesthesia	84 (22.1)
	Allodynia	11 (2.9)
	Anesthesia	5 (1.3)
Neurological signs	Sensory disturbance	277 (72.9)
	Muscle weakness	277 (72.9)
	Areflexia	234 (61.6)
	Hyporeflexia	111 (29.2)
	Atrophy	93 (24.5)
	Sensory ataxia	90 (23.7)
	Romberg sign	83 (21.8)
	Foot and leg abnormalities^d^	58 (15.3)
	Hypotonia	46 (12.1)

n: number of patients who presented each variable; ^a^Cyclosporine, chloroquine, RIPE regimen for tuberculosis, phenytoin, methotrexate, chemotherapy, radiotherapy, tacrolimus, thalidomide, antiretroviral therapy for HIV, treatment for leprosy; ^b^Chemical agents (thinner, methylene oxide, lead paint), pesticides, cocaine, and crack; ^c^Hepatitis B and hepatitis C; ^d^Claw toes, hammer toes, nonspecific deformities of hands and feet, pes cavus, equine foot, flat foot, leg in champagne bottle. EDX: Electrodiagnostic.

Table 2 summarizes the etiology of the polyneuropathy in the sample population. The majority of patients (75.2%) had one etiology for polyneuropathy (monocausal group), 11.1% had two or more concomitant etiologies (multifactorial group), and 13.7% had no etiological factor identified (idiopathic group). 

**Table 2.** Classification of the study group according to etiology.

Etiology		n (%)	
Monocausal polyneuropathies	Inflammatory	90 (23.7)	286 (75.2)
	Hereditary	72 (18.9)	
	Diabetes	41 (10.8)	
	Vasculitis	17 (4.5)	
	Toxicity^a^	17 (4.5)	
	Alcohol abuse	14 (3.7)	
	Infection^b^	12 (3.2)	
	Nutritional deficiency ^c^	11 (2.9)	
	Metabolic^d^	4 (1.1)	
	Malignancy	4 (1.1)	
	Critical illness	4 (1.1)	
Multifactorial polyneuropathies	Diabetes and hypothyroidism	6 (1.5)	42 (11.1)
	Alcohol abuse and vitamin B12 deficiency	5 (1.3)	
	Diabetes and vitamin B12 deficiency	4 (1.1)	
	HIV and antiretroviral therapy	4 (1.1)	
	Diabetes and toxic medication^a^	4 (1.1)	
	Diabetes and alcohol abuse	3 (0.7)	
	Alcohol and infectious disease^b^	2 (0.5)	
	Diabetes and infectious disease^b^	2 (0.5)	
	Alcohol abuse and toxic medication^a^	2 (0.5)	
	Other combinations	10 (2.6)	
Idiopathic polyneuropathies	Complete basic investigation	37 (9.7)	52 (13.7)
	Incomplete basic investigation	15 (3.9)	
Total		380	

n: number of cases for each polyneuropathy etiology; ^a^Toxic medication (chloroquine, stavudine, phenytoin, chemotherapy, radiotherapy, RIPE regimen for tuberculosis, tacrolimus, thalidomide, antiretroviral therapy for HIV, treatment for leprosy), contact with pesticides, and contact with chemical agents (methylene oxide, thinner, lead paint); ^b^HIV or leprosy; ^c^Vitamin B12 deficiency or vitamin E deficiency; ^d^Acute intermittent porphyria or hypothyroidism.

In general, considering the entire sample of the study rather than each group individually, the five main causes of polyneuropathy confirmed by EDX testing were inflammatory, hereditary, idiopathic, multifactorial, and diabetic polyneuropathy.

From the group of monocausal polyneuropathies, the three main etiologies were inflammatory, hereditary, and diabetes. Inflammatory etiology occurred in 23.7% of the patients: 16.3% occurred by GBS (and its variants AMAN, AMSAN, and Miller-Fischer syndrome), 4.7% by CIDP, and 2.6% by other causes (including multifocal motor neuropathy and Lewis-Sumner syndrome). The median age of patients with GBS was 33 years (range 2-76) and 46 years (range 2-81) for those with CIDP. In both cases, there was a predominance of males (74.2% in GBS and 66.7% in CIDP). A hereditary etiology was diagnosed in 18.9% of patients, with a median age of 13 years (range 0.3-75) and a predominance in males (61.1%). Charcot-Marie-Tooth was the main cause of hereditary polyneuropathy, corresponding to 10.2% of the total sample. The third most common isolated etiology was diabetes, accounting for 10.8% of the investigated patients. The median age for the diabetic etiology was 55 years (range 22-77) and, unlike the first two causes, there was a predominance in females (56.1%). The frequency of the other etiologies is shown in Table 2.

Multifactorial polyneuropathy was diagnosed in 42 patients (11.1%). Diabetes was the most involved etiology, being present in half of the cases. The most frequent combination was diabetes and hypothyroidism (Table 2). 

In 13.7% of the patients, no etiological cause of peripheral neuropathy was identified, being classified as idiopathic polyneuropathy, either by complete basic investigation (9.7%) or incomplete basic investigation (3.9%). The median age was 51.5 years (range 1-85) for idiopathic polyneuropathy with incomplete basic investigation and 45.5 years (range 15-79) for those with complete basic investigation. In both groups, there was a similar distribution between male and female individuals.

Considering risk factors, 88 patients had diabetes, 40 of whom developed diabetic polyneuropathy, 21 multifactorial polyneuropathy, 10 inflammatory polyneuropathy (7 with GBS, 2 with CIDP and 1 with Lewis-Sumner syndrome), and 17 patients presented with different etiologies. A similar situation occurred with alcohol abuse: 54 patients had a current or previous history of alcoholism, but only 14 patients were diagnosed with alcoholic polyneuropathy. Thirteen patients were diagnosed with multifactorial polyneuropathy, 5 were classified as inflammatory polyneuropathy (4 with GBS and 1 with CIDP), another 5 as hereditary polyneuropathy, and 17 had different etiologies, as did diabetic patients. The presence of different etiologies occurred because these patients did not fulfilled the criteria for diabetic or alcoholic polyneuropathy or because there was a lack of temporal correlation.

**Figure 2.**
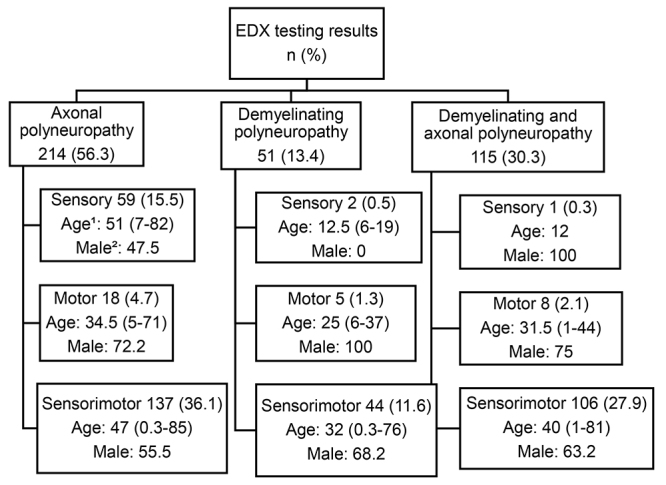
Flow chart of polyneuropathies epidemiological profile according to neurophysiological aspects.

**Figure 3.**
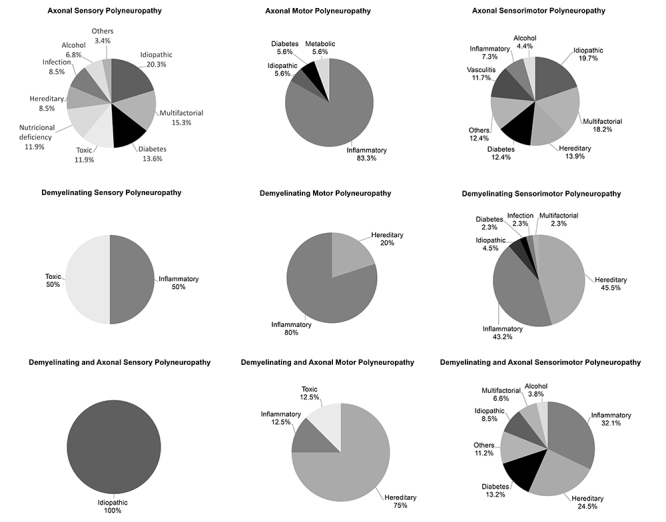
Distribution of different polyneuropathies according to the neurophysiological study.

Figure 2 shows the frequency of each electrophysiological pattern, as well as median age and gender distribution. Figure 3 shows the main causes for each electrophysiological pattern. The main electrophysiological patterns were axonal sensorimotor polyneuropathy (36.1%) and “demyelinating and axonal” sensorimotor polyneuropathy (27.9%). Individuals with demyelinating polyneuropathies had a lower median age than those with axonal forms. Compared with demyelinating and “demyelinating and axonal” patterns, axonal patterns showed greater etiological heterogeneity, with a predominance of idiopathic and multifactorial polyneuropathy. However, in axonal motor polyneuropathies, there was a predominance of the inflammatory etiology. Analyzing demyelinating and “demyelinating and axonal” polyneuropathies, there was a lower number of etiologies, with a predominance of hereditary and inflammatory polyneuropathies. 

n: number of cases of each neurophysiological pattern; EDX: electrodiagnostic test; ^1^median age (range minimum -maximum); ^2^percentage of male patients**.**

## DISCUSSION

The five main causes of polyneuropathy confirmed by EDX testing were inflammatory, hereditary, idiopathic, multifactorial, and diabetic polyneuropathy. From these data, our study indicates that polyneuropathies submitted to electrodiagnostic testing are those recently advocated by the AANEM in its official position: polyneuropathies that show a severe, atypical, rapidly progressing course, with predominant motor involvement, a positive family history, or when no cause is identified despite initial assessment^9^. 

From the EDX tests performed on suspicion of polyneuropathy, 48.9% confirmed this condition and 17.7% revealed a different diagnosis. This corroborates the fact that EDX not only confirms polyneuropathy but also detects other associated neuromuscular diseases and reveals other diagnoses, specially mononeuropathies and radiculopathies. Ginsberg and Morren recently found similar results^28^; the EDX of patients with suspected diabetic polyneuropathy presented an alternative diagnosis in about 20% of patients, and in 25% of those patients, it detected other overlapping diseases. Thus, it would be interesting to explore polyneuropathies due to common causes to better determine the origin of symptoms and the outcome^28^^,^^29^.

In our study, the frequencies of the identified etiologies differ from other studies conducted in tertiary hospitals^8^^,^^30^^-^^34^. The reason for this probably lies in the fact that our study used a retrospective design with the objective of identifying the main causes of polyneuropathies confirmed by electrophysiological study from a specialized center, with the selection of patients based on EDX. Epidemiological studies are usually prospective, selecting patients according to symptomatology and risk factors, and only later performing nerve a conduction study, for which abnormal EDX is not an inclusion criterion^1^.

Inflammatory polyneuropathy had a frequency of approximately 24%, which is higher than previously described^8^^,^^30^^-^^34^. The frequency was similar to the results of Rudolph and Farbu, who identified inflammatory polyneuropathy as the main cause of monocausal polyneuropathy in 24% of individuals^27^. GBS (and its variants) and CIDP were the main conditions in this etiological group, which was characterized by the predominance of demyelinating electrophysiological alterations^35^^,^^36^. Axonal motor polyneuropathy was also related to inflammatory polyneuropathy, mainly due to the diagnosis of one of the GBS variants (e.g., AMAN).

Idiopathic polyneuropathy occurred in 14% of the patients in our sample, a pattern that is different from some epidemiological studies in specialized hospitals, which have reported frequencies of 20 to 49%^8^^,^^27^^,^^30^^,^^31^^,^^33^. The results are similar to Lin et al. and Verghese et al., who demonstrated idiopathic polyneuropathy frequencies of 12 and 13%, respectively^32^^,^^34^. Indeed, a decreasing incidence of idiopathic polyneuropathy has already been reported due to the improvement in the diagnosis of lower prevalence polyneuropathies through more sophisticated diagnostic guidelines^37^^,^^38^. In our study, idiopathic polyneuropathy was the main cause of axonal sensory and axonal sensorimotor polyneuropathies, with a median age of around 50 years, in accordance with previous literature data^37^^,^^38^. 

The frequency of diabetic polyneuropathy (around 11%) was lower than in previous studies, which reported frequencies ranging from 20 and 50%^1^^,^^4^. This divergence can be explained by several factors. In addition to the aforementioned divergence in methodology, most epidemiological studies do not classify polyneuropathies as multifactorial, as in this study. Of the multifactorial polyneuropathy cases, 50% presented diabetes as one of the etiological factors. Another explanation for this lower occurrence is the fact that only patients with severe diabetic polyneuropathy were referred to our center. Mild and moderate forms of diabetic polyneuropathy, included in population-based studies, are not usually submitted to EDX tests in our center.

Because this study was conducted in a specialized center, a possible selection bias of patients in the sample might have occurred, with a predominance of severe polyneuropathies. Hence, the results cannot be extrapolated to the general population. Another limitation of this study is the non-inclusion of patients with normal EDX tests. Although the objective did not include the evaluation of small fiber polyneuropathy, it is known that patients with a clinical picture of polyneuropathy and normal nerve conduction studies may present large fiber dysfunction with abnormalities in the EDX test during the course of the disease^15^^,^^16^. Thus, it is necessary to consider a possible underestimation of our frequencies.

On the other hand, in the present study, cases of polyneuropathy were submitted to EDX tests and different etiologies were found in each neurophysiological pattern. Therefore, when exploring whether the pathophysiology is axonal or demyelinating, EDX testing guides differential diagnosis of rare and atypical polyneuropathies. Our results indicate that axonal polyneuropathies result from idiopathic, infectious, toxic, nutritional deficiency, vasculitic, and metabolic causes, while demyelinating polyneuropathies have predominantly inflammatory and hereditary causes, in accordance to the literature^9^^,^^39^ It is important to identify these etiologies because some of them, such as inflammatory and vasculitic causes, have disease-modifying therapies, including corticosteroids and immunoglobulin^9^^,^^39^.In conclusion, polyneuropathies in this study were predominantly from inflammatory, hereditary, idiopathic, multifactorial, and diabetic causes. It is clear that polyneuropathies confirmed by EDX testing in a specialized center are those that most challenge clinical reasoning because they are atypical, hereditary, severe, require rapid management, or because there is no diagnostic clue in the medical history and laboratory tests. By understanding how neurophysiological patterns correlate with specific etiologies and that the electrophysiological study can reveal other diagnoses, we can deduce that EDX testing is useful for the etiological diagnosis of less common polyneuropathies and contributes to the initiation of correct and early treatment, avoiding inappropriate treatments, with possible gain in quality of life for the patient.
